# Increased Production of Outer Membrane Vesicles by Salmonella Interferes with Complement-Mediated Innate Immune Attack

**DOI:** 10.1128/mBio.00869-21

**Published:** 2021-06-01

**Authors:** Ruchika Dehinwal, Danielle Cooley, Alexey V. Rakov, Akhil S. Alugupalli, Joey Harmon, Olivier Cunrath, Prashanth Vallabhajosyula, Dirk Bumann, Dieter M. Schifferli

**Affiliations:** a Department of Pathobiology, University of Pennsylvania, School of Veterinary Medicine, Philadelphia, Pennsylvania, USA; b Department of Surgery, Hospital of the University of Pennsylvania, Philadelphia, Pennsylvania, USA; c Biozentrum, University of Basel, Basel, Switzerland; University of Washington

**Keywords:** *Salmonella*, *S.* Typhimurium, PagC, Rck, outer membrane vesicles, PhoPQ, C3b, Factor H, complement resistance

## Abstract

Bacterial outer membrane vesicles (OMVs) enriched with bioactive proteins, toxins, and virulence factors play a critical role in host-pathogen and microbial interactions. The two-component system PhoP-PhoQ (PhoPQ) of Salmonella enterica orchestrates the remodeling of outer membrane lipopolysaccharide (LPS) molecules and concomitantly upregulates OMV production. In this study, we document a novel use of nanoparticle tracking analysis to determine bacterial OMV size and number. Among the PhoPQ-activated genes tested, *pagC* expression had the most significant effect on the upregulation of OMV production. We provide the first evidence that PhoPQ-mediated upregulation of OMV production contributes to bacterial survival by interfering with complement activation. OMVs protected bacteria in a dose-dependent manner, and bacteria were highly susceptible to complement-mediated killing in their absence. OMVs from bacteria expressing PagC bound to complement component C3b in a dose-dependent manner and inactivated it by recruiting complement inhibitor Factor H. As we also found that Factor H binds to PagC, we propose that PagC interferes with complement-mediated killing of Salmonella in the following two steps: first by engaging Factor H, and second, through the production of PagC-enriched OMVs that divert and inactivate the complement away from the bacteria. Since PhoPQ activation occurs intracellularly, the resultant increase in PagC expression and OMV production is suggested to contribute to the local and systemic spread of Salmonella released from dying host cells that supports the infection of new cells.

## INTRODUCTION

Salmonella enterica is a Gram-negative bacterial pathogen that can survive and replicate in both phagocytic and nonphagocytic cells (1, 2) thanks in part to its two-component system PhoP and PhoQ, designated PhoPQ (3). PhoPQ is activated by low Mg^2+^, acidic pH, and cationic antimicrobial peptides in Salmonella-containing vacuoles and regulates the expression of genes required for intracellular survival (4–6), in part by activating the SPI-2 type III secretion system (T3SS) (3). Activation of PhoPQ also induces the expression of genes encoding outer membrane proteins (OMPs), as well as regulators and enzymes that modify outer membrane (OM) components. Recapitulation of the shift from extracellular to intracellular environmental conditions *in vitro* leads to activation of the *phoPQ* regulon and covalent modification of OM lipopolysaccharides (LPSs), thereby destabilizing the highly cross-linked OM (7). These changes increase outer membrane vesicle (OMV) formation and help in the removal of negatively charged LPS detrimental for intracellular survival and in its replacement with modified LPS that is more neutral (7). Accordingly, constitutive expression or induction of PhoPQ-*a*ctivated *g*enes (*pag*s), such as *pagP* or *pagL* which encode OM enzymes that add or remove acyl groups from LPS, result in increased OMV production and concomitant removal of charged LPS, while deletion of these genes reduced the production of OMVs under different experimental conditions (8, 9).

OMVs are spherical (20- to 200-nm diameter) membranous structures primarily composed of LPSs, phospholipids, OMPs, and a lumen filled with cargo that consist mainly of periplasmic proteins (10). OMVs play critical roles in bacterium-bacterium and bacterium-host interactions (11). The production of OMVs allows the bacterium to interact with its environment and mediate pathogenesis through biofilm formation, horizontal gene transfer, intra- and interspecies communication, delivery of toxins, killing of competing microbial cells, resistance to antibiotics, adherence to host cells, and immunomodulation (10, 12–16). Since the proteins of various PhoPQ-activated genes can play a role in OMV production (8, 9, 17), we undertook a systematic analysis of OMVs made by deletion mutants of PhoPQ-regulated genes. We used nanoparticle tracking analysis (NTA) to compare the size and number of OMVs produced. Several mutants showed reduced OMV production, but deletion of *pagC* had the most significant effect. Thus, we further investigated the role of PagC in the formation of OMVs and assessed the potential implications of these PagC-induced OMVs in Salmonella pathogenesis.

PagC belongs to a family of integral OMPs that form a barrel-shaped transmembrane structure with 8 β-strands and 4 extracellular loops (18, 19). It plays a role in biofilm formation and shares homology with OMPs such as Rck encoded on a Salmonella enterica serovar Typhimurium plasmid and OmpX/Ail of various *Enterobacteriaceae* (20–27). While both Rck and Ail mediate serum resistance (23, 28–32), the ability of PagC to provide S. Typhimurium with the same protection remains controversial in the literature (33, 34). Therefore, we investigated a possible link between either PagC or OMV production and serum resistance. Notably, we identified a novel PagC-dependent mechanism by which Salmonella evades complement-mediated bacterial killing. Specifically, we described a role for PagC in upregulating OMV production and further demonstrated that OMVs produced by PagC-expressing bacteria attract complement component C3b and inactivate it by recruiting Factor H. As PagC is enriched in OMVs and binds to Factor H, we propose that OMVs triggered by PagC expression serve as complement decoys that trap and inactivate C3b, protecting Salmonella from the bactericidal effect of serum, thereby aiding in local and systemic spread.

## RESULTS

### PagC is an activator of OMV production.

**FIG 1:**
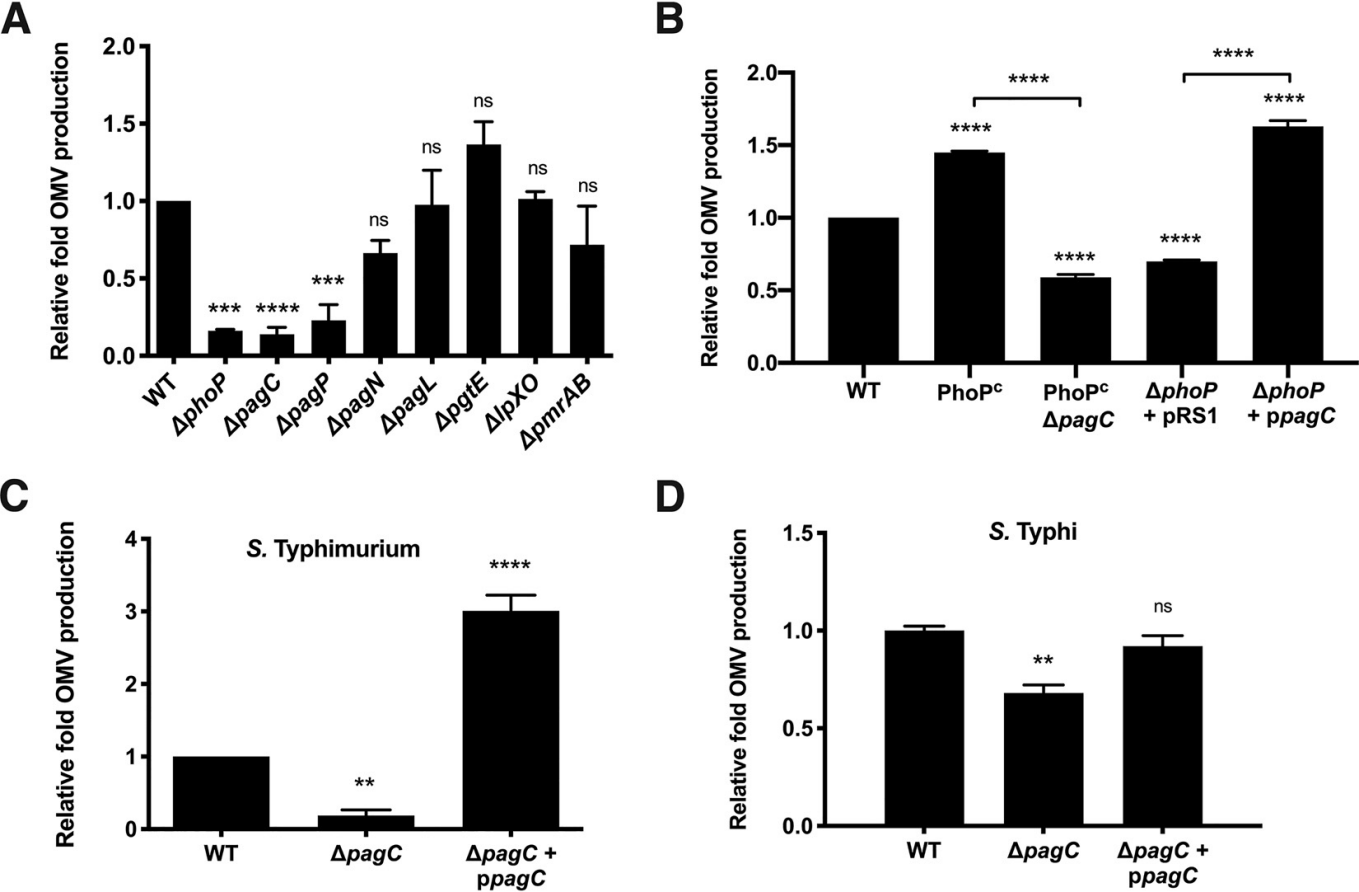
Quantitative analysis of OMV production by Salmonella under PhoPQ-activating conditions (5.8L). All S. Typhimurium strains used were flagella mutants (*fliC fljB*). (A) Relative fold OMV production by 2 × 10^9^ CFU/ml bacteria was calculated relative to the OMVs produced by parental strain S. Typhimurium *fliC fljB* (labeled as WT) normalized to 1. NTA analysis showed that the OMV production was significantly downregulated in Δ*phoP*, Δ*pagC*, and Δ*pagP* mutants. (B) A constitutively active *phoP* mutant (PhoP^C^) and the Δ*phoP* mutant induced to express PagC from plasmid p*pagC* showed a hypervesiculating phenotype. Deletion of *pagC* in PhoP^C^ and the Δ*phoP* mutant containing the empty vector (pRS1) decreased OMV production by ∼3-fold. (C) The Δ*pagC* mutants of S. Typhimurium and S. Typhi showed significant reductions in OMV production compared with their respective WT parental strains. The phenotype of the S. Typhi strain could be restored by complementing the Δ*pagC* mutants with plasmid p*pagC* induced to express PagC. The data represent one of three separate, reproducible experiments, expressed as mean ± SEM. Statistical significance was calculated using one-way ANOVA multiple-comparison test with significance set at a *P* value of <0.05 (*, *P < *0.05; **, *P < *0.01; ***, *P < *0.001; ****, *P < *0.0001; and ns, *P > *0.05).

The Salmonella PhoPQ regulator senses the host environment to promote OM remodeling, during which enzymes are activated to modify a “new” LPS, while the “old” unmodified LPS is recycled via OMV production (7). To identify which PhoPQ-activated genes are involved in enhancing OMV production, we isolated OMVs from various *pag* deletion mutants grown under PhoPQ-activating conditions (5.8L N-minimal media). The number of OMVs as enumerated by NTA decreased by 6.2-fold for Δ*phoP*, 4.3-fold for Δ*pagP*, and 7.3-fold for Δ*pagC* mutants compared with the parental strain S. Typhimurium *fliC fljB*, designated wild type (WT) in this study (Fig. 1A; see Table S1 in the supplemental material). In contrast, no such significant differences were found in the deletion mutants of other PhoPQ-activated genes, including *pagN*, *pagL*, *pgtE*, *lpxO*, and *pmrAB*. Deletion of *pagC* in a strain that constitutively expressed *phoP* (PhoP^C^ Δ*pagC*) reduced the number of OMVs by 2.4-fold compared with the PhoP^C^ mutant, whereas plasmid-based induction of PagC (p*pagC*) in a Δ*phoP* mutant increased the number of OMVs produced by 3.2-fold compared with the Δ*phoP* mutant containing just the empty vector (Fig. 1B and Table S1). We also measured the size (diameter) of the OMVs by both transmission electron microscopy (TEM) (see Fig. S1A in the supplemental material) and NTA (Fig. S1B). The NTA analysis revealed that the diameter of OMVs was not significantly changed by deletion of any of the *pag*s studied (*P* > 0.05 for each mutant compared with WT) (Fig. S1C). To determine if the effects of *pagC* on OMV production were recapitulated in a Salmonella serovar that is more virulent for humans, we generated a deletion mutant of *pagC* in Salmonella enterica serovar Typhi. This mutant also showed a hypovesiculating phenotype compared with the wild-type strain (Fig. 1D; Table S1). Complementation of PagC expression by the p*pagC* vector in a Δ*pagC* mutant restored the vesiculation phenotype in both S. Typhi and S. Typhimurium (Fig. 1C and D; Table S1). Taken together, the data indicated that PagC is a significant activator of OMV production in different Salmonella serovars.

10.1128/mBio.00869-21.1 FIG S1 WT and Δ*pagC* OMVs as analyzed by TEM (A) and nanoparticle tracking analysis (B). (A) Average diameter of OMVs for WT was 138.94 ± 86 nm (mean ± SD) and 114.38 ± 57 nm for Δ*pagC*, as determined from TEM micrographs and measured by NIH ImageJ software. (B) Peak represents the modal value of the experimental data (154 nm or 149 nm for WT and Δ*pagC* OMVs, respectively), as calculated by NTA analysis. (C) Mean size (diameter in nm) of OMVs produced by Salmonella grown under PhoPQ-activating conditions, as measured by NTA analysis. The data represent one of three separate, reproducible experiments, expressed as mean ± SEM. Statistical significance was calculated using the one-way ANOVA multiple-comparison test (ns, *P > *0.05). Download FIG S1, PDF file, 1.9 MB. Copyright © 2021 Dehinwal et al. 2021 Dehinwal et al. https://creativecommons.org/licenses/by/4.0/ This content is distributed under the terms of the Creative Commons Attribution 4.0 International license.

10.1128/mBio.00869-21.2 TABLE S1 Number of OMVs/ml from various Salmonella deletion mutants. Download Table S1, DOCX file, 0.02 MB. Copyright © 2021 Dehinwal et al. 2021 Dehinwal et al. https://creativecommons.org/licenses/by/4.0/ This content is distributed under the terms of the Creative Commons Attribution 4.0 International license.

To determine whether the level and/or repertoire of OMPs in OMVs were affected in the Δ*pagC* mutant, we undertook a comparative mass spectrometry analysis of OMPs in OMVs from WT and Δ*pagC* bacteria. OMPs detected in the OMVs of the SL1344, ATCC14028s, and LT2 (35) wild-type strains were essentially comparable (see Table S2 in the supplemental material). Using OmpA as the baseline standard for OMPs, we found that the relative abundance of most detected OMPs in the OMVs from the Δ*pagC* mutant only varied slightly compared with those in OMVs from the WT strain (Table S2). However, the relative abundance of some ion transport proteins, such as FoxA, CirA, and FepA, increased significantly in the Δ*pagC* mutant, as did the porins OmpF and OmpC, the surface receptors FepE and IroN, and the LPS-assembly lipoprotein (LptE). In addition, the relative amounts of FadL, Blc, and the OMP-assembly factor BamA were decreased in the Δ*pagC* mutant. Thus, the absence of PagC dysregulated OMV production not only by reducing their generation but also by modulating the ratios of selected OMPs.

10.1128/mBio.00869-21.3 TABLE S2 Outer membrane proteins (OMPs) detected in OMVs from the WT strain and the Δ*pagC* mutant. Download Table S2, XLSX file, 0.02 MB. Copyright © 2021 Dehinwal et al. 2021 Dehinwal et al. https://creativecommons.org/licenses/by/4.0/ This content is distributed under the terms of the Creative Commons Attribution 4.0 International license.

**FIG 2:**
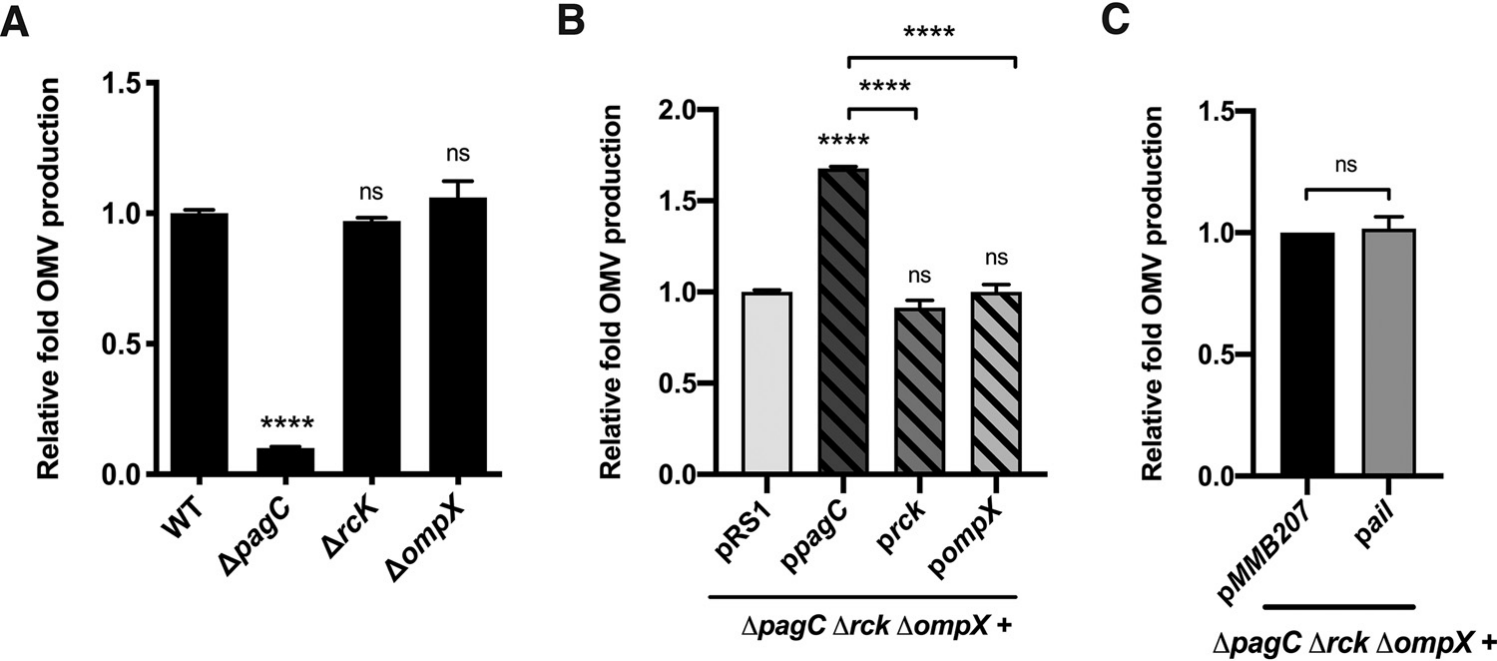
Relative fold OMV production by Salmonella grown under PhoPQ-activating conditions (5.8L). (A) Relative fold OMV production by the Δ*pagC* mutant was significantly reduced compared with WT bacteria or Δ*rck* or Δ*ompX* mutants. Deletion of *rck* or *ompX* had no significant effect on OMV production. (B) OMVs produced by the Δ*pagC* Δ*rck* Δ*ompX* triple mutant expressing PagC, Rck, or OmpX from corresponding plasmids showed that OMV production is upregulated only by the PagC-expressing plasmid in the Δ*pagC* Δ*rck* Δ*ompX* triple mutant. Plasmid pRS1 is the control empty vector. The data represent one of three separate, reproducible experiments, expressed as mean ± SEM. (C) Relative fold OMV production by Δ*pagC* Δ*rck* Δ*ompX* triple mutant containing empty vector p*MMB207* or p*ail* induced to express Ail from Y. pestis (100 μM isopropyl-β-d-thiogalactopyranoside [IPTG]) showed no significant change in OMV production. Statistical significance was calculated using one-way ANOVA multiple-comparison test and set at a *P* value of <0.05 (****, *P < *0.0001; and ns, *P > *0.05).

PagC shares sequence similarity with two other S. Typhimurium OMPs, namely, RcK (54%) and OmpX (36%), raising the possibility that they could play a role in OMV production. However, deletion of either *rck* or *ompX*, of which both encode OMPs present in OMVs of the wild-type strain (Table S2), did not alter the number of OMVs produced under PhoPQ-activating conditions (Fig. 2A and Table S1). Furthermore, in contrast to PagC, plasmid-driven expression of S. Typhimurium Rck or OmpX did not alter OMV production in a Δ*pagC* Δ*rck* Δ*ompX* triple deletion mutant (Fig. 2B and Table S1). Additionally, the OMP Ail of Yersinia pestis, which is also similar to PagC (36%), did not affect OMV production when expressed from a plasmid in the Δ*pagC* Δ*rck* Δ*ompX* triple mutant (Fig. 2C and Table S1). Thus, only PagC among the set of PagC-similar bacterial OMPs (Rck, OmpX, and Ail) activates OMV production.

### Salmonella OM protein PagC mediates resistance to serum-dependent bacterial killing.

**FIG 3:**
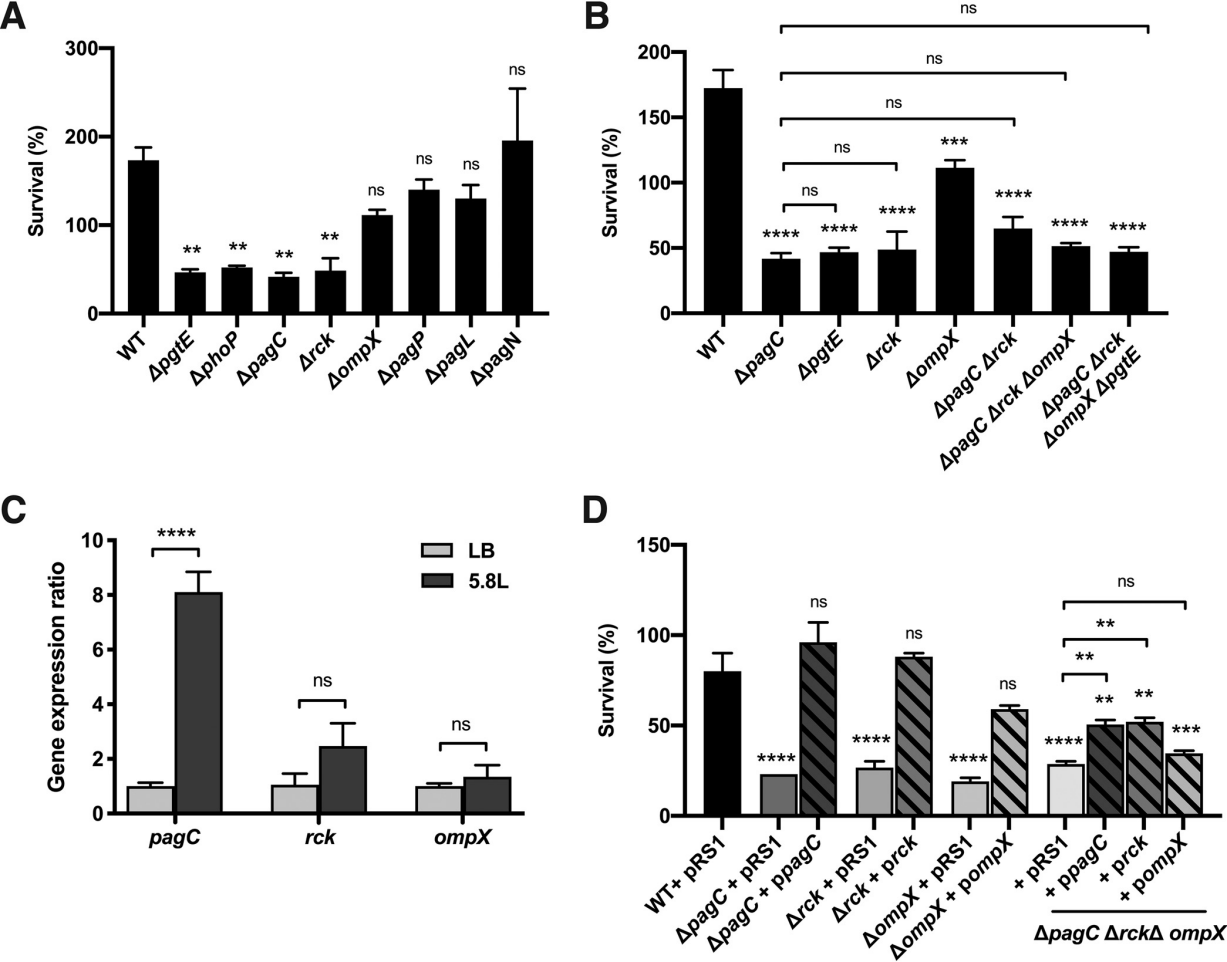
Serum resistance assay for Salmonella mutants grown under PhoPQ-activating conditions. (A) Survival of Salmonella deletion mutants in 25% NHS incubated at 37°C for 1 h. Deletion of Δ*pgtE*, Δ*rck* (used as positive controls), Δ*phoP*, and Δ*pagC* made the bacteria susceptible to complement-mediated killing. (B) A comparison of serum resistance between Salmonella Δ*pagC*, Δ*rck*, and Δ*ompX* single, double, and triple mutants. (C) Quantitative RT-PCR-based analysis of gene expression ratio of *pagC*, *rck*, and *ompX* (relative to housekeeping gene *rpoB*) in wild-type Salmonella grown in LB or under PhoPQ-activating conditions (5.8L) showed the expression of *pagC* increased significantly under PhoPQ-activating conditions. (D) Resistance to serum-mediated attack was restored in single Salmonella mutants complemented with plasmids induced to express PagC, Rck, and OmpX in the corresponding mutants. Plasmids expressing Rck or PagC complemented best the triple mutant compared with mutants containing empty vector (pRS1). The data represent one of three separate, reproducible experiments, expressed as mean ± SEM. Statistical significance was calculated using one-way ANOVA multiple-comparison test and set at a *P* value of <0.05 (*, *P < *0.05; **, *P < *0.01; ***, *P < *0.001; ****, *P < *0.0001; and ns, *P > *0.05).

The sequence similarity of PagC with other OMPs, such as the S. Typhimurium Rck and Y. pestis Ail, that provide serum resistance prompted us to reevaluate the role of PagC in resistance to serum-mediated bacterial killing, an issue plagued by conflicting results in the literature (33, 34). For this evaluation, we incubated S. Typhimurium WT and various isogenic deletion mutants of PhoPQ-activated genes with 25% normal human serum (NHS) and compared their resistance to serum-mediated killing. Deletions of *pagC* and *phoP* made bacteria more susceptible to serum-mediated killing, as did deletions of the *pgtE* or *rck* gene, which are both known to inactivate serum/complement-mediated bacterial killing and serve as PhoPQ-dependent and PhoPQ-independent positive controls, respectively (36, 37). In contrast, deletion of other PhoPQ-activated genes, such as *pagP*, *pagL*, and *pagN*, had no significant effect on bacterial survival (Fig. 3A). The Ail-similar OmpX of Salmonella was only weakly protective (Fig. 3A). Notably, the relative abundance of Rck, OmpX, and PgtE in OMVs from the Δ*pagC* mutant remained comparable to OMVs from the WT strain (0.8 to 1.7 times) (Table S2), indicating that these proteins were unable to compensate for the absence of PagC in inhibiting serum-mediated killing.

To determine if PagC, Rck, and OmpX mediate resistance to complement attack in an additive manner, we compared the serum resistance activity of double and triple mutants with that of the corresponding single mutants (Fig. 3B). Surprisingly, the triple and double mutants were essentially as susceptible as the single mutants, indicating the absence of additive effects under the experimental conditions used. Some level of resistance remained with all the interrogated mutants, as well as a quadruple mutant that included *pgtE*, suggesting the presence of other potentially protective bacterial components.

Since the absence of additive effects could be related to the suboptimal expression of *rck* and *ompX* under PhoPQ-activating conditions, we compared the mRNA levels of *pagC*, *rck*, and *ompX* in Salmonella grown in LB and under 5.8L conditions. Notably, *pagC* expression alone was significantly upregulated under 5.8L conditions, with the expression of the other two genes being only slightly higher than that in LB (Fig. 3C). However, as the absence of one of the genes could potentially influence the expression of the others, we next used plasmids to individually induce PagC, Rck, or OmpX expression in the single and triple mutants (Fig. 3D). Complementation of the single mutants confirmed that each protein can individually interfere with serum killing effects. In the triple mutant, PagC or Rck expressed from plasmids could compensate for the absence of the other proteins to interfere with serum killing, albeit at a lower level than that in the single complemented mutants or wild-type strain. Thus, in contrast to the data obtained with noncomplemented mutants (Fig. 3B), complementation of the triple mutant with plasmids expressing just one of the proteins suggested potential additive effects of their activities (Fig. 3D). The OmpX-expressing plasmid did not complement the triple mutant, which is in agreement with the weaker effect of serum on the single *ompX* mutant. Taken together, the data indicated that the protective effect of PagC, Rck, and OmpX depends on their individual level of expression. As expression levels are most likely determined by environmental signals, our findings suggest that each of these proteins uniquely provide bacterial protection in nonoverlapping host compartments.

### PhoPQ-induced OMVs interfere with complement-mediated killing of Salmonella in a dose-dependent manner.

**FIG 4:**
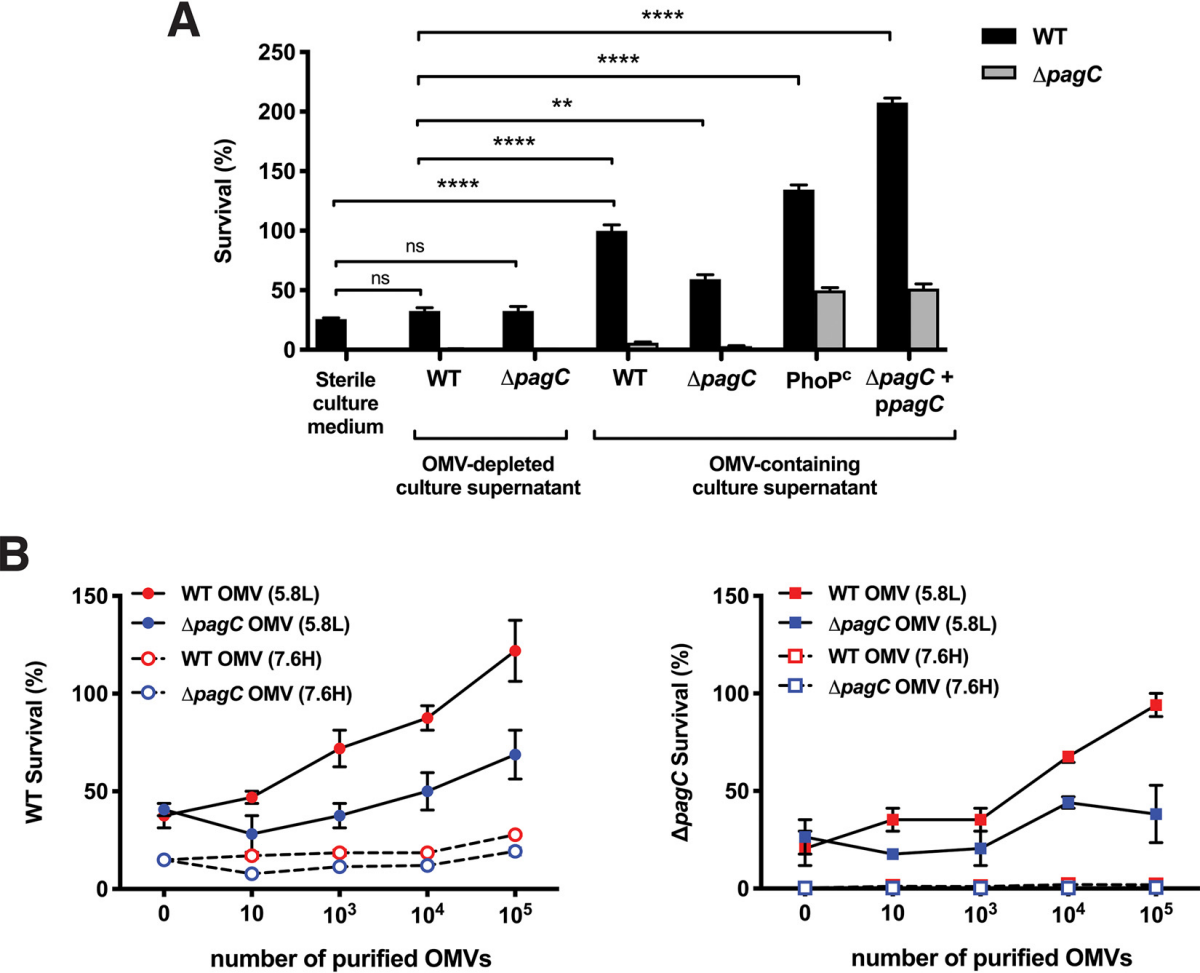
Serum protection assay by OMV produced under PhoPQ-activating conditions. (A) Survival of WT and Δ*pagC* Salmonella incubated with 25% NHS in the presence of sterile culture medium, OMV-depleted culture supernatants, or OMV-containing culture supernatants showed that bacteria incubated with OMV-containing culture supernatants from PagC-expressing bacteria (WT, Δ*pagC* + p*pagC*, and PhoP^C^) were significantly more resistant to serum-mediated attack than bacteria incubated with sterile culture medium or culture supernatant from the Δ*pagC* mutant. Bacterial survival in sterile culture medium was comparable to the survival in OMV-depleted spent culture supernatants. (B) Survival of WT and Δ*pagC* Salmonella incubated with 25% NHS in the presence of purified OMVs at indicated concentrations (0 to 10^5^ OMVs), isolated from bacteria grown under PhoPQ-activating (5.8L; solid line) or nonactivating (7.6H; dashed line) conditions. OMVs from bacteria expressing PagC (WT) grown under PhoPQ-activating conditions were more protective than OMVs from bacteria grown under nonactivating conditions. The data represent one of three separate, reproducible experiments, expressed as mean ± SEM. Statistical significance was calculated using Student’s *t* test and set at a *P* value of <0.05 (*, *P < *0.05; **, *P < *0.01; ***, *P < *0.001; ****, *P < *0.0001; and ns, *P > *0.05).

To determine if OMVs induced by PagC activation play a role in resistance to serum-mediated killing, OMV-free WT Salmonella and an isogenic Δ*pagC* mutant bacteria were resuspended in either sterile culture medium or OMV-containing spent culture supernatants from WT, Δ*pagC*, Δ*phoP*, PhoP^C^, or Δ*pagC* p*pagC* mutants before incubating with 25% NHS (Fig. 4A). Bacteria incubated without OMVs were susceptible to serum-mediated killing, whereas the bacteria incubated with OMV-containing culture supernatants from any Salmonella were significantly more resistant to the attack (*P < *0.01). The OMV-free WT bacteria supplemented with OMV-containing culture supernatants from the PhoP^C^ or Δ*pagC* p*pagC* strains, which overexpress *pagC*, were significantly more serum resistant than when supplemented with their own OMV-containing culture supernatant, while OMV-containing culture supernatants from the Δ*pagC* mutant protected significantly less efficiently. Comparable results were obtained with the isogenic Δ*pagC* mutant (Fig. 4A, gray bars), albeit each separate OMV addition protected significantly less efficiently than in the WT strain (Fig. 4A, black bars). To rule out the potential influence of a hypothetical component from the spent culture supernatants on serum-mediated attack, OMV-free WT bacteria were resuspended in OMV-containing or OMV-depleted spent culture supernatants, before incubating with 25% NHS. The bacteria were significantly better protected when resuspended in OMV-containing spent culture supernatants than in OMV-depleted spent culture supernatants (Fig. 4A). Moreover, bacterial survival was essentially the same when bacteria were resuspended in either sterile culture media or OMV-depleted spent culture supernatants, suggesting that bacterial protection against complement attack was essentially only due to the presence of OMVs.

To bypass confounding effects due to variable OMV numbers in the different spent culture supernatants, we undertook further experiments using equal numbers of purified OMVs prepared from bacteria grown under conditions that do or do not activate PhoPQ (Fig. 4B). Only purified OMVs from bacteria produced under PhoPQ-activating conditions interfered with serum killing of WT bacteria in a dose-dependent manner, with the OMVs from the WT strain being significantly more protective than OMVs from the Δ*pagC* mutant. The Δ*pagC* mutant bacteria were protected in a dose-dependent manner only by OMVs from the WT strain produced under PhoPQ activation. Taken together, the data confirmed that under the PhoPQ-activating conditions, OMVs were most protective against serum when they were produced from Salmonella that express PagC.

### PhoPQ-induced OMVs block the AP by mediating C3b degradation.

**FIG 5:**
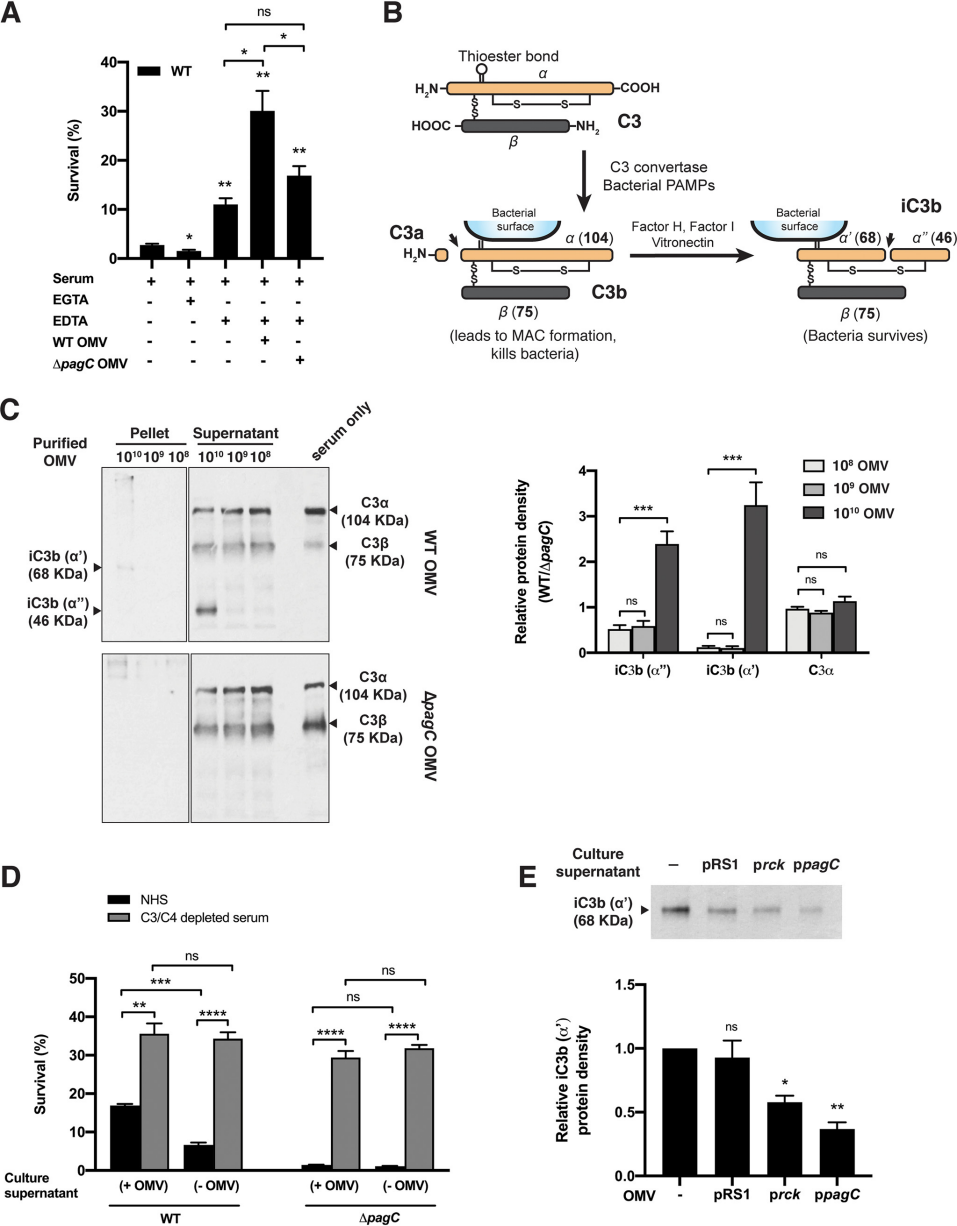
Blocking of complement activation by OMVs from PagC-expressing bacteria. (A) Survival of WT Salmonella incubated with 25% NHS treated with 5 mM EGTA or EDTA, in the presence or absence of 1 × 10^9^ purified OMVs from WT or Δ*pagC* mutant. OMVs from PagC-expressing WT bacteria block the alternative pathway of the complement more efficiently than the OMVs from the Δ*pagC* mutant. Statistical significance was calculated using Student’s *t* test. (B) Schematic representation of C3 and its cleavage products C3b and iC3b (molecular weight [MW] of the α and β chain and the cleaved or uncleaved products is indicated in the brackets). (C) WT OMVs (10^8^ to 10^10^ OMVs) incubated with 5% NHS, when immunoblotted with anti-C3 antibody, showed bands for C3b inactivation at 68 kDa (iC3bα′) and 46 kDa (iC3bα″) in OMV pellet and supernatant fractions, respectively. No C3b inactivation bands were found when Δ*pagC* OMVs were incubated with NHS, suggesting that WT OMVs bind to C3b and inactivate C3b into iC3b in a dose-dependent manner. The Western blot image was spliced to remove an empty lane in between pellet and supernatant fractions. The panel on the right represents relative protein band densities (WT versus Δ*pagC* OMVs) as analyzed by NIH ImageJ software from 3 separate, reproducible experiments, expressed as mean ± SEM. Statistical significance was calculated using Student’s *t* test. (D) WT or Δ*pagC* mutant incubated with C3/C4-depleted human serum (gray bars) survived significantly better than the bacteria incubated with NHS (black bars). Presence of OMV-containing culture supernatant (+ OMV) contributed to bacterial survival when incubated with NHS, compared with bacteria incubated in the presence of OMV-depleted culture supernatants (− OMV). (E) Incubation of the Δ*pagC* Δ*rck* Δ*ompX* Δ*pgtE* mutant with NHS in the presence of OMV-containing culture supernatant prevented C3b deposition on bacterial cell surfaces. OMVs from the quadruple mutant expressing PagC prevented C3 deposition most significantly. The bar graph below the blot represents relative protein band densities as analyzed by NIH ImageJ software from 3 separate, reproducible experiments, expressed as mean ± SEM. For iC3bα′, relative protein densities were calculated for bacteria incubated in sterile culture medium (−) with no OMVs versus bacteria incubated with OMV-containing spent culture supernatant. Statistical significance was calculated using the one-way ANOVA multiple-comparison test. The data represent one of three separate, reproducible experiments, expressed as mean ± SEM (*, *P < *0.05; **, *P < *0.01; ***, *P < *0.001; ****, *P < *0.0001; and ns, *P > *0.05).

Rck blocks both the alternative pathway (AP) and classical pathway (CP) of the complement by binding to complement inhibitors (29, 31). To identify the complement pathways that are blocked by OMVs, OMV-free Salmonella were reconstituted with purified OMVs from the WT or Δ*pagC* mutant strains (1 × 10^9^ OMVs) and incubated with human serum treated with EGTA (to block the CP and lectin pathways) or EDTA (to block all 3 complement pathways). As shown in Fig. 5A, WT bacteria without any OMVs were eight times more susceptible to killing when incubated with serum treated with EGTA (AP active) than to the bacteria incubated with EDTA-treated serum (AP blocked). The effects of the AP were also blocked by incubating the bacteria in the presence of OMVs, and the importance of *pagC* was highlighted by the relatively higher level of protection provided by OMVs from WT bacteria than OMVs from the Δ*pagC* mutant.

Since the C3 component of the AP is activated upon interaction with bacterial surface molecules such as LPS (38), we investigated whether OMVs also activate C3. For this investigation, purified WT or Δ*pagC* mutant OMVs were incubated with 5% NHS and analyzed by immunoblotting with anti-human C3 antibody as described in Materials and Methods. The presence of a 68-kDa (iC3bα′) band in the OMV pellet fraction and a 46-kDa (iC3bα″) band in the supernatant fraction of WT OMVs but not Δ*pagC* mutant OMVs (Fig. 5C) suggested that OMVs from PagC-expressing bacteria bind to and degrade complement component C3b into iC3bα′ and iC3bα″, the typical fragments of C3b inactivation (Fig. 5B) (39). A decrease in the strength of a band at 104 kDa (C3α) with an increasing number of purified WT OMVs showed that OMVs from PagC-expressing bacteria degrade the complement in a dose-dependent manner. Incubation of the Salmonella WT strain and the Δ*pagC* mutant with 25% NHS or C3/C4-depleted human serum in the presence of OMV-containing culture supernatant revealed that both strains survived significantly better when incubated with C3/C4-depleted serum and that this enhanced survival was independent of the presence of OMVs (Fig. 5D). As the level of bacterial survival in NHS was OMV dependent, these results further confirm that OMVs interfere with complement-mediated killing of bacteria in a C3-dependent manner. Incubation of the Δ*pagC* Δ*rck* Δ*ompX* Δ*pgtE* quadruple mutant with NHS in the presence of sterile culture medium showed a significantly larger amount of C3b (68 kDa) associated with bacterial pellets relative to the bacteria incubated in the presence of OMV-containing culture supernatant from the quadruple mutant induced to express PagC or Rck (Fig. 5E). Taken together, our data suggest that the interaction of C3b with OMVs from PagC-expressing bacteria syphons off and inactivates C3b from the serum, thereby interfering with the bactericidal properties of serum.

### OMVs from PagC-expressing bacteria recruit complement inhibitor Factor H.

**FIG 6:**
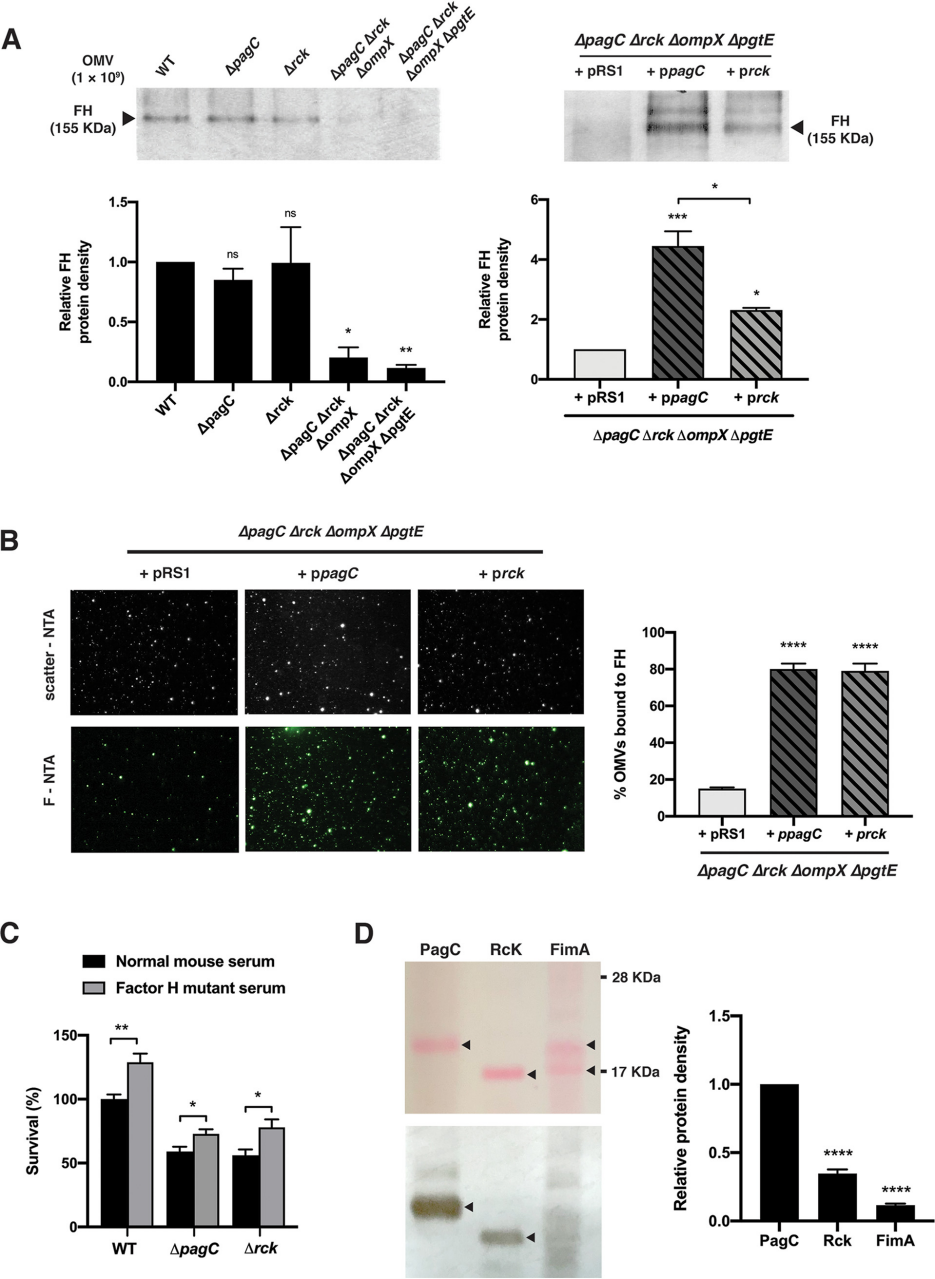
Factor H interaction with OMVs. (A) 1 × 10^9^ Purified OMVs from WT, Δ*pagC*, Δ*rck*, Δ*pagC* Δ*rck* Δ*ompX*, or Δ*pagC* Δ*rck* Δ*ompX* Δ*pgtE* mutant bacteria incubated with purified Factor H (FH; 2 μg) showed FH binding by OMVs from PagC- or Rck-expressing bacteria, as detected by Western blotting using anti-FH antibody. The bar graphs below each blot represent relative protein band density as analyzed by NIH ImageJ software from 3 separate, reproducible experiments, expressed as mean ± SEM. The relative protein density was calculated relative to WT bacteria (left) or the Δ*pagC* Δ*rck* Δ*ompX* Δ*pgtE* + pRS1 (empty vector)-containing mutant (right). (B) F-NTA analysis showed a significantly high percentage of OMVs from the PagC- or Rck-expressing quadruple mutant bound to FH compared with OMVs from the pRS1-containing quadruple mutant. The % OMVs bound to FH were calculated as the number of OMVs estimated by F-NTA versus the number of OMVs estimated by scatter-NTA × 100. (C) Incubation of WT, Δ*pagC* mutant, or Δ*rck* mutant bacteria with normal mouse serum or FH mutant (W1206R) serum. Both PagC and Rck contribute to bacterial survival in serum in an FH-dependent manner. Statistical significance was calculated using Student’s *t* test (A and C). (D) Far-Western analysis of FH binding to recombinant proteins PagC, Rck, and FimA (1 μg each, indicated by arrowheads) showed purified FH (1 μg/ml) bound only to PagC and Rck, with the former showing greater affinity for FH. FimA being only partially reduced shows two bands in the blot stained by Ponceau S. Molecular weight standards are indicated on the right. The graph on the right represents relative protein densities as analyzed by the NIH ImageJ software, averaging 3 separate, reproducible experiments (mean ± SEM). The protein band densities for Rck and FimA were calculated relative to the PagC protein. Statistical significance was calculated using the one-way ANOVA multiple-comparison test (B and D). The data represent one of three separate, reproducible experiments, expressed as mean ± SEM (*, *P < *0.05; **, *P < *0.01; ***, *P < *0.001; ****, *P < *0.0001; and ns, *P > *0.05).

The ability of OMVs from PagC-expressing bacteria to degrade complement component C3b suggests that OMVs can recruit complement inhibitors such as Factor H (FH) or vitronectin. As shown in Fig. 6A, when purified OMVs of Salmonella strains were incubated with purified FH, a significant association was observed, with OMVs from the strains expressing PagC and/or Rck. OMVs from the quadruple Δ*pagC* Δ*rck* Δ*ompX* Δ*pgtE* mutant complemented to express PagC bound significantly more FH than OMVs from the same mutant induced to express Rck (Fig. 6A and B). Moreover, when purified OMVs from the quadruple mutant bacteria were incubated in solution with purified FH and analyzed by fluorescence-NTA (F-NTA), significantly fewer OMVs from empty vector-containing bacteria bound FH (15%) than OMVs from p*pagC-* or p*rck-*carrying bacteria (80% and 79%, respectively) (Fig. 6B; see Fig. S4 to S9 in the supplemental material). These results indicated not only that PagC and Rck present surface-accessible domains on OMVs but also that these domains mediate FH binding to OMVs. In contrast, no significant binding of vitronectin to OMVs could be detected (data not shown). Incubation of WT or Δ*pagC* or Δ*rck* mutant bacteria with normal mouse serum or FH mutant (W1206R) mouse serum (40) showed that PagC and Rck each contribute independently to bacterial survival in serum in an FH-dependent manner that is additive with the WT strain (Fig. 6C). Furthermore, a Western blot experiment with PagC (18 kDa), Rck (17 kDa), and FimA (18 kDa, type 1 fimbrial subunit used as control) recombinant proteins showed that FH bound to both PagC and Rck, but not FimA, confirming the specific recruitment of FH by the two former proteins (Fig. 6D).

## DISCUSSION

The complement system provides a formidable first line of immune attack against any invading pathogenic microbe. Therefore, not surprisingly, many pathogens have evolved various arsenals of defense mechanisms to protect themselves against soluble molecules of the complement system and its attack complex (41). In this study, we demonstrated that the OMP PagC allows S. Typhimurium to evade complement-mediated killing by two distinct mechanisms; it promotes the production of PagC-enriched OMVs that serve as complement traps and also inactivates C3b by recruiting the complement inhibitor Factor H.

In this study, we took advantage of corroborative NTA instruments to quantitatively measure bacterial OMV number and demonstrated that S. Typhimurium SL1344 upregulates OMV production upon PhoPQ activation. We also demonstrated with a single *pagC* deletion mutant and *trans*-complementation that PagC alone is a key inducer of OMV production (Fig. 1A). Unlike the PhoPQ-induced PagP and PagL enzymes that are thought to activate OMV production by modifying the acylation profile of LPS and membrane curvature (8, 9, 42–44), PagC, which is transcribed and produced in much larger amounts after PhoPQ activation (35, 45), has no known LPS-modifying properties and can induce Salmonella vesiculation by itself.

A previous report showed that PagC expression under PhoPQ activation induces OMV production in a Δ*clpXP* S. Typhimurium, a mutant that lacks the ATP protease ClpXP (17). Counterintuitively, and in contrast to our findings, the authors also demonstrated that a Δ*clpXP* Δ*pagC* deletion mutant did not affect vesiculation. However, ClpXP targets degradation of the alternative sigma factor RpoS, which coordinates the expression of nearly 23% of Salmonella small RNAs, with each regulating the expression of various proteins, including the major porin OmpD (17, 46–52). Thus, the authors proposed that the *clpXP* deletion altered OMV production in a manner that might have obscured the *pagC* deletion phenotype (17). However, this conclusion warrants further investigation, particularly since the authors also mentioned that OMV numbers were not reduced in a single *pagC* deletion mutant (17), without further discussion of this finding.

In 1994, Heffernan et al. cloned and expressed a *pagC* gene of S. Typhimurium in Escherichia coli HB101 and showed that it was not required for resistance to serum-mediated bacterial killing (33). In contrast, a later study with S. enterica serovar Choleraesuis by Nishio et al. demonstrated that PagC confers a high level of serum resistance when cloned into E. coli or Salmonella strains (34). The reason for this discrepancy remains unclear since both studies followed essentially the same protocol using S. Typhimurium or S. Choleraesuis *pagC* genes that encode the same allelic protein. In the present study, we confirmed the observation by Nishio et al., and additionally, we established that PagC interferes with the lethal effects of the human complement toward Salmonella in two synergistic ways.

The ability of PhoPQ-activated PagC to promote Salmonella resistance to complement attack is directly linked, in part, to its ability to induce production of OMVs, which protect bacteria in a dose-dependent manner. We hypothesized that OMVs from PagC-expressing bacteria act as decoys and prevent complement deposition and activation on the bacterial cell surface. In support, we showed that the incubation of OMVs from PagC-expressing bacteria with OMV-free Salmonella significantly reduced C3 deposition on the bacterial cell surface. We also showed that bacterial survival was significantly higher when they were incubated with C3/C4-depleted serum, confirming that bacterial killing by NHS was complement dependent. In addition, PagC itself directly interfered with the alternative complement pathway. We demonstrated that OMVs from PagC-expressing bacteria bind to soluble complement component C3b and recruit complement inhibitor Factor H to inactivate C3b into iC3b (Fig. 5C and 6A, respectively), thus preventing C3 convertase formation. This mechanism interferes with the opsonization of Salmonella and blocks the amplification of the complement pathway that would lead to the formation of the membrane attack complex responsible for bacterial lysis (41).

Interestingly, even though our experiments confirmed that Rck, an OMP homologous to PagC, inhibits complement activity (28, 29, 31), unlike PagC, Rck had no effect on Salmonella vesiculation. Similarly, Salmonella OmpX and Y. pestis Ail proteins, additional PagC-like proteins investigated here, did not affect OMV production, and the OmpX was significantly less protective than PagC or Rck against the bactericidal property of serum. Other bacteria, such as Vibrio cholerae, make OMVs with OMPs that can trap and inactivate complement factors, but these OMPs do not activate OMV production (53, 54). Since PagC seems uniquely able to promote OMV production, it will be important to determine the mechanistic basis for this effect. Given the role of LPS in OMV formation (8) and the reported structural interactions of LPS and the PagC-similar Ail OMP (18), it will be particularly informative to identify the residues of PagC that interact with LPS and determine their potential impact on OMV formation.

Despite the strong inhibitory effects of PagC on serum-mediated killing, OMVs from the Δ*pagC* mutant still offered some protection, suggesting the potential involvement of alternative protective mechanisms. Additional OMPs, such as Rck, OmpX, PgtE, and TraT, can contribute to Salmonella resistance to the host complement (36, 37, 55). However, the amounts of Rck, OmpX, PgtE, and TraT were comparable or slightly increased in purified OMVs from the Δ*pagC* mutant versus wild type OMVs, and OMVs from the Δ*pagC* mutant were only weakly protective. While it remains possible that the reduced abundance of other OMPs, such as PagN, in the Δ*pagC* mutant (Table S2) contributed to the reduced protective property of the OMVs of this mutant, the pagN mutation was not associated with reduced OMV production (Fig. 1A) or bacterial susceptibility (Fig. 3A). These results suggested that PagC is a key contributor of interference with serum-mediating killing for PhoPQ-activated Salmonella. We confirmed a PgtE effect on serum killing and OMV production but did not investigate TraT, as like Rck, TraT is plasmid encoded and thus absent in strains and serovars that lack this plasmid. Whether TraT alone or yet another surface molecule was responsible for the detected residual survival of bacteria in serum remains to be determined. In addition, S. Typhimurium PhoPQ indirectly activates enzymes that modulate the chain length and complexity of the LPS O-antigen (56, 57), sterically hindering formation of the C9-membrane attack complex on the bacterial surface (58), which may also contribute to the residual protection.

The number of Salmonella surface molecules involved in complement resistance suggests a strong evolutionary selection for protection against this major extracellular pathogen-killing mechanism in host tissues, particularly following inflammation with vascular leakage of serum. Following Salmonella-induced cell death, the bacteria leave the intracellular compartment, either directly from the vacuole or by transiting first through the cytoplasmic compartment. Even though the presence of Salmonella in the extracellular environment might be brief compared with its intracellular residence in hosts, Salmonella remains highly susceptible to complement attack in such an environment. Thus, we propose that the production of protective molecules differentially induced by various regulons ensures that Salmonella is preconditioned for serum resistance in all relevant host compartments, whether Salmonella cycles between host phagocytic cells or through intestinal epithelial cells (59–61). Indeed, our results confirmed that PagC is induced by signals specific to intravacuolar conditions, whereas Rck is upregulated in response to homoserine lactone (62), a quorum sensing signal typically ascribed to the induction of biofilm formation. Thus, Rck likely protects Salmonella where it can multiply to high local density, such as an extravasate-covered intestinal lumen surface. In contrast, PagC induction of OMVs following PhoPQ activation is suggested to provide a cover of protection for the bacteria when they cycle through host cells after being released with their OMVs from dying cells. Specifically, as we demonstrated that Factor H binds PagC, we propose that OMVs released from dying cells bind to and deactivate C3b by recruiting Factor H, thereby promoting further infection by permitting extracellular bacteria to thwart complement-mediated death. The intravacuolar induction of OMV production to prepare Salmonella for the next environment is reminiscent of an evolutionary mechanism of bacterial survival that has been described in the literature as an anticipatory strategy (60, 61).

Few studies have yet investigated the potential role of PagC in bacteria serum resistance *in vivo*. In one study, a Salmonella *pagC-*Tn*phoA* mutant that was administered orally or intraperitoneally (i.p.) to BALB/c mice revealed a reduced 50% lethal concentration (LD_50_) compared with that of the parental wild-type strain (63). However, this mutant was later shown to be attenuated due to the production of a fusion protein, leaving the original question unresolved (64). A second study using competition experiments with wild-type and Δ*pagC* mutant Salmonella injected simultaneously into mice did not demonstrate any significant effect (65). However, it is likely that the wild-type strain with its large number of OMVs may have masked any potential Δ*pagC* mutant phenotype linked to reduced vesiculation.

The important effects of PagC on OMV production and complement evasion clearly warrant further noncompetitive *in vivo* studies for determining its impact on pathogenesis. Notably, Salmonella-infected patients generate antibodies to PagC (66), which would also be expected to be captured by OMVs. Thus, potential therapeutic PagC inhibitors designed to block OMV production could likely compromise Salmonella traps of both host innate and adaptive immune molecules, further highlighting their utility in reducing the pathogenicity of these enteric and systemic bacteria.

## MATERIALS AND METHODS

### Bacterial strains and culture conditions.

All bacterial strains, including mutants and plasmids used in this study are listed in Table S3, along with references 77–82. Unless stated otherwise, all the reagents were procured from MilliporeSigma (St. Louis, MO, USA). The Salmonella strains were grown at 37°C in Luria-Bertani (LB) broth or N-minimal medium [5 mM KCl, 7.5 mM (NH_4_)_2_SO_4_, 0.5 mM K_2_SO_4_, 1 mM KH_2_PO_4_, and 0.1% Casamino Acids] with 0.4% glucose and supplemented with 10 μM MgSO_4_ (buffered with 0.1 M morpholineethanesulfonic acid [MES; pH 5.8]; for PhoPQ-activating, 5.8L conditions) or 10 mM MgSO_4_ (buffered with 0.1 M morpholinepropanesulfonic acid [MOPS; pH 7.6]; for 7.6H conditions), (7). To grow Salmonella under PhoPQ-activating conditions, bacteria were grown in LB and then sequentially transitioned into 7.6H and 5.8L conditions. A S. Typhimurium SL1344 defective in the production of flagellins (*fliC* and *fljB*) was prepared by generalized transduction with phage P22, and this strain (designated wild type [WT]) was used as the parental background strain to engineer all Salmonella deletion mutants. Deletion of *fliC* and *fljB* was done to avoid the interference of flagella with OMV purification and analysis. The Salmonella deletion mutants were prepared using Gibson assembly and allelic exchange methods, essentially as described previously (67, 68). The mutants were analyzed for growth defects by growing the bacteria in LB and N-minimal media. No significant difference in growth was found between the wild type or any deletion mutant when adapted to a corresponding medium condition. The primers used in this study are listed in Table S3. To constitutively express PhoPQ-activated genes (PhoP^C^) in S. Typhimurium SL1344, a *phoP-* and *phoQ-*containing amplicon flanking 1,000 bp upstream and downstream of the *phoP* gene was amplified from a ATCC14028s *pho*-24 S. Typhimurium mutant strain containing the PhoQ T48I mutation (69) and cloned into a SL1344 Δ*phoP* mutant by Gibson assembly and allelic exchange methods. For *trans*-complementation of protein expression, the genes were amplified from S. enterica serovar Typhimurium SL1344 genomic DNA and cloned in a pRS1 plasmid using the Gibson assembly master mix (New England BioLabs Inc., Ipswich, MA, USA). The amplicons were cloned into plasmid pGM81Δ*envZ* using Gibson assembly and induced for expression with anhydrotetracycline hydrochloride (AHT; 0.4 μg/ml) for 2 h at 37°C, starting with log-phase cultures (A_600_ = 0.3). When appropriate, antibiotics were used at the following concentrations: ampicillin at 200 μg/ml, kanamycin at 25 μg/ml, chloramphenicol at 30 μg/ml, and streptomycin at 90 μg/ml.

10.1128/mBio.00869-21.4 TABLE S3 List of bacterial strains, plasmids, and primers. Download Table S3, DOCX file, 0.03 MB. Copyright © 2021 Dehinwal et al. 2021 Dehinwal et al. https://creativecommons.org/licenses/by/4.0/ This content is distributed under the terms of the Creative Commons Attribution 4.0 International license.

### OMV purification.

Salmonella cultures grown overnight in 5.8L or 7.6H N-minimal media were centrifuged with a Beckman Avanti J-E centrifuge at 15,000 × *g* (JLA-16.250 rotor) for 10 min at 4°C to obtain bacterial cell pellets and OMV-containing culture supernatants. The culture supernatant was centrifuged again at 15,000 × *g* (JLA-16.250 rotor) for 10 min at 4°C and filtered through 0.45-μm cellulose membrane filters to remove any bacterial cell and cellular debris contamination (70–72). Filtered supernatants were then subjected to ultracentrifugation in a Beckman optima L-90K ultracentrifuge at 300,000 × *g* (Type 50.2 Ti rotor) for 90 min at 4°C to pellet OMVs. The obtained OMV pellets were resuspended in sterile phosphate-buffered saline (PBS), filtered through a 0.2-μm filter, and checked for bacterial contamination by plating onto LB agar before storing at −20°C for future use.

### OMV characterization.

The concentration and size (diameters) of OMVs, normalized by CFU/ml, were quantified by transmission electron microscopy (TEM) and by nanoparticle tracking analysis (NTA) using NanoSight NS300 (Malvern Panalytical, Malvern, UK) or ZetaView (Particle Matrix, Meerbusch, Germany) instruments at the Extracellular Vesicle Core, University of Pennsylvania. For TEM, purified OMVs were placed on 300-mesh copper grids (Electron Microscopy Sciences Hatfield, PA, USA), stained with 1% uranyl acetate for 30 sec, and viewed using FEI Tecnai T12 electron microscope at the EMRL facility of the University of Pennsylvania. The diameter of OMVs observed by TEM analysis was calculated by using the measurement function in the NIH ImageJ v. 1.53 software (http://rsbweb.nih.gov/ij/index.html) for 45 fields per strain at a magnification of 1:100.000. Differences in OMV sizes measured by TEM and NTA can be attributed to overestimations of vesicle size by NTA devices (73).

### Proteomic profiling of OMPs.

Identification of OMPs present in the OMVs of the WT strain and Δ*pagC* mutant was determined by mass spectrometry-based analysis using a Thermo Scientific Orbitrap Fusion instrument, paired to a Thermo Scientific Ultimate 3000 ultra-high-performance liquid chromatography (UHPLC), at the Quantitative Proteomics Research Core facility, University of Pennsylvania. The collected tandem mass spectrometry (MS/MS) spectra were analyzed against the UniProt database of S. enterica serovar Typhimurium SL1344 and compared to OMV-associated OMPs (acidic conditions) from ATCC14028s and LT2 OMVs (35). The abundance of the identified OMPs in the OMVs of the Δ*pagC* mutant and WT Salmonella strain were each normalized to the abundance of OmpA in the respective strains to calculate a relative abundance for each OMP (Δ*pagC* versus WT, Table S2).

### Serum resistance assays.

For analyzing the sensitivity of Salmonella to serum-mediated killing, bacteria (∼1 × 10^5^ CFU grown in 5.8L N-minimal media (optical density at 600 nm [OD_600_], 0.5) were incubated with 25% normal human serum (NHS) (Astarte Biologics, Bothell, WA, USA), C3/C4-depleted human serum (Complement Technology Inc., TX, USA), or with normal mouse serum and Factor H mutant serum (40) for 1 hour at 37°C. The number of viable bacteria after incubation was calculated by plating serial dilutions on LB agar. To analyze the role of OMVs in resistance to complement-mediated killing, OMV-free bacteria were obtained by washing the bacteria (grown to an OD_600_ of 0.5) three times with sterile PBS to remove OMVs. Spent culture supernatant from bacteria grown under 5.8L conditions (OD_600_, 0.5) was filtered through a 0.2-μ filter to obtain OMV-containing spent culture supernatants or ultracentrifuged to remove OMVs (OMV-depleted spent culture supernatants). The OMV-free bacteria (∼1 × 10^5^ CFU) were resuspended in either sterile culture medium, OMV-depleted, or OMV-containing spent culture supernatants in an equivalent volume ratio. Some experiments were done with OMV-free bacteria incubated with purified OMVs resuspended at specific concentrations in PBS. The resuspended bacteria were then incubated with 25% NHS at 37°C, and the number of viable bacteria after 1 h of incubation was calculated by plating onto LB agar plates. To block CP and LP of the complement, NHS was supplemented with 5 mM EGTA and 10 mM MgCl_2_ or with 5 mM EDTA to block all 3 pathways (CP, LP, and AP).

### Quantitative RT-PCR Assay.

Quantitative reverse transcription-PCR (RT-PCR) was performed as described previously (74). Briefly, total mRNA from the bacterial strains grown in LB or 5.8L N-minimal media (approximately OD_600_ of 0.5) was extracted using the TRIzol reagent (Gibco BRL, Waltham, MA, USA) according to the manufacturer’s protocol. Residual genomic DNA contamination was removed by treatment with DNase, Turbo DNA-free kit (Ambion, Austin, TX, USA), and the RNA was checked for purity by agarose gel electrophoresis. An absence of DNA contamination was confirmed by PCR without reverse transcription, and RNA concentration was determined with the ND1000 spectrophotometer (NanoDrop products, Wilmington, DE, USA). Four hundred nanograms of RNA was used in reverse transcription reactions using random hexamer primers and a high-capacity cDNA reverse transcription kit (Applied Biosystems, Foster City, CA, USA) according to the manufacturer’s protocol. Quantitative real-time PCR was carried out using cDNA corresponding to 20 ng RNA, gene-specific primers (Table S3), and the PowerUP SYBR green PCR master mix (Applied Biosystems) with the ABI Fast 7500 real-time PCR system (Applied Biosystems). Expression levels were normalized to the transcription levels for the housekeeping gene *rpo*B, and relative copy numbers were calculated according to the threshold cycle (2^−ΔΔ^*^CT^*) method.

### Binding of OMVs to complement components.

OMVs (10^8^ to 10^10^ OMVs) isolated from bacteria grown in 5.8L N-minimal media were incubated with 5% NHS or 2 μg of purified human Factor H protein (Complement Technology Inc., TX, USA) for 1 hour at 37°C. The mixture was then centrifuged at 200,000 × *g* (Type 42.2 Ti rotor) for 15 min at 4°C to collect the OMV pellet and the supernatant. The supernatant (containing unbound complement components) and the OMV pellet (containing OMV-bound complement components) were washed twice with sterile PBS and analyzed by Western blotting using horseradish peroxidase (HRP)-conjugated anti-human C3 antibody (1:5,000) (MP Biomedicals, Irvine, CA, USA) or anti-human Factor H antibody (1:500) (Complement Technology Inc., TX, USA). Donkey anti-goat HRP-conjugated antibody at a 1:10,000 dilution was used as the secondary antibody (Jackson ImmunoResearch, PA, USA) followed by probing the blots with ECL substrate (GE Healthcare Life Sciences, MA, USA). Alternatively, interference of C3 deposition on the bacterial cell surface by OMVs was detected by incubating OMV-free Δ*pagC* Δ*rck* Δ*ompX* Δ*pgtE* mutant bacteria resuspended in either sterile culture medium or OMV-containing culture supernatants of the Δ*pagC* Δ*rck*Δ *ompX* Δ*pgtE* mutant carrying plasmids induced to express Rck or PagC with 25% NHS for 1 hour at 37°C. The bacteria were then pelleted and washed twice with PBS to remove OMVs and unbound complement. Complement deposited on bacterial cell surfaces was detected by Western blot analysis of cell lysates using the HRP-conjugated anti-human C3 antibody. Binding of vitronectin to OMV-free WT Salmonella (1 × 10^7^ CFU in the presence of PBS or purified WT or Δ*pagC* OMVs [1 × 10^9^]) was analyzed by incubating the mixture with 25% NHS for 1 hour at 37°C. The mixture was then centrifuged to collect supernatant, bacterial pellet (15,000 × *g*, 10 min, 4°C), and OMV pellet (200,000 × *g*, 15 min, 4°C). Vitronectin binding to the bacteria and OMVs was investigated by Western blotting using anti-human vitronectin (1:500; Complement Technology Inc.) and HRP-conjugated secondary antibody as described above. The images generated from Western blotting were subjected to densitometric analysis by NIH ImageJ software.

### Far-Western and Immunolabelling.

A total of 1 μg of recombinant Salmonella PagC, Rck, and FimA proteins (75) were subjected to 15% SDS-PAGE. Proteins were electrotransferred onto a nitrocellulose membrane and blocked with 3% bovine serum albumin (BSA). Subsequently, the membrane was incubated with purified human FH (1 μg/ml, in 0.1% BSA), which is below the physiological concentration of FH in serum (500 μg/ml) (76). Binding of the protein was detected by immunoblotting with goat anti-human FH and donkey anti-goat HRP-conjugated secondary antibody as described above. For immunolabelling the OMVs, purified OMVs from the quadruple mutant induced to express Rck or PagC from corresponding plasmids were incubated with 2 μg of purified human FH, in a final volume of 200 μl. The OMVs were then washed with sterile PBS at 200,000 × *g* (Type 42.2 Ti rotor) for 15 min at 4°C followed by incubation with anti-human Factor H antibody (1:1,000 dilution) and a 1:2,000 diluted donkey anti-goat Alexa Fluor-488 antibody (Abcam, Cambridge, UK) as the labeled secondary antibody. The fluorescently labeled OMVs were then analyzed by fluorescence NTA (F-NTA) using Zetaview.

### Statistics.

One-way analysis of variance (ANOVA) multiple comparisons or Student’s *t* test were used for statistical calculations using Prism v. 8 (GraphPad Software, San Diego, CA, USA). Statistical significance was set at a *P* value of <0.05 (*, *P < *0.05; **, *P < *0.01; ***, *P < *0.001; and ****, *P < *0.0001).

10.1128/mBio.00869-21.5 VIDEO S1 Scatter-NTA of OMVs from quadruple mutant (Δ*pagC* Δ*rck* Δ*ompX* Δ*pgtE*) containing empty vector (pRS1). Download Movie S1, MOV file, 6.1 MB. Copyright © 2021 Dehinwal et al. 2021 Dehinwal et al. https://creativecommons.org/licenses/by/4.0/ This content is distributed under the terms of the Creative Commons Attribution 4.0 International license.

10.1128/mBio.00869-21.6 VIDEO S2 Scatter-NTA of OMVs from quadruple mutant expressing PagC (p*pagC*). Download Movie S2, MOV file, 6.1 MB. Copyright © 2021 Dehinwal et al. 2021 Dehinwal et al. https://creativecommons.org/licenses/by/4.0/ This content is distributed under the terms of the Creative Commons Attribution 4.0 International license.

10.1128/mBio.00869-21.7 VIDEO S3 Scatter-NTA of OMVs from quadruple mutant expressing Rck (p*rck*). Download Movie S3, MOV file, 6 MB. Copyright © 2021 Dehinwal et al. 2021 Dehinwal et al. https://creativecommons.org/licenses/by/4.0/ This content is distributed under the terms of the Creative Commons Attribution 4.0 International license.

10.1128/mBio.00869-21.8 VIDEO S4 Fluorescence-NTA (F-NTA) showing binding of FH to OMVs from quadruple mutant containing empty vector (pRS1). Download Movie S4, MOV file, 2.1 MB. Copyright © 2021 Dehinwal et al. 2021 Dehinwal et al. https://creativecommons.org/licenses/by/4.0/ This content is distributed under the terms of the Creative Commons Attribution 4.0 International license.

10.1128/mBio.00869-21.9 VIDEO S5 F-NTA showing binding of FH to OMVs from quadruple mutant expressing PagC (p*pagC*). Download Movie S5, MOV file, 6.2 MB. Copyright © 2021 Dehinwal et al. 2021 Dehinwal et al. https://creativecommons.org/licenses/by/4.0/ This content is distributed under the terms of the Creative Commons Attribution 4.0 International license.

10.1128/mBio.00869-21.10 VIDEO S6 F-NTA showing binding of FH to OMVs from quadruple mutant expressing Rck (p*rck*). Download Movie S6, MOV file, 6.1 MB. Copyright © 2021 Dehinwal et al. 2021 Dehinwal et al. https://creativecommons.org/licenses/by/4.0/ This content is distributed under the terms of the Creative Commons Attribution 4.0 International license.
