# Supplementary figures and images for: Increased Production of Outer Membrane Vesicles by Salmonella Interferes with Complement-Mediated Innate Immune Attack
Source: mBio. 2021 Jun 1;12(3):e00869-21. doi: 10.1128/mBio.00869-21 (PMC8262969; doi:10.1128/mBio.00869-21)

**A**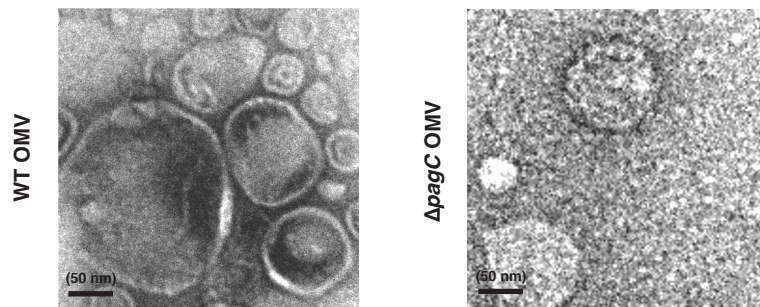**B**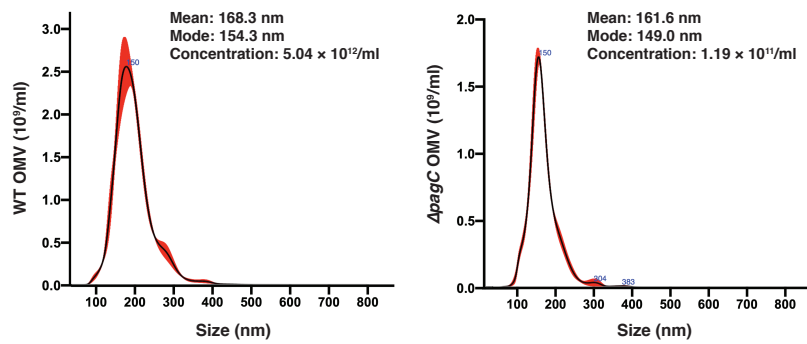**C**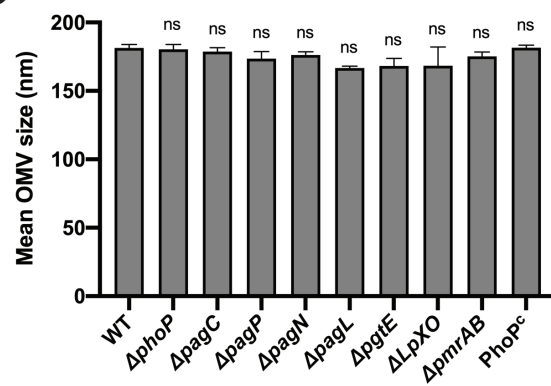

Supplement: FIG S1 [file mbio.00869-21-sf001.pdf]
