# Supplementary material for: Increased Production of Outer Membrane Vesicles by Salmonella Interferes with Complement-Mediated Innate Immune Attack
Source: mBio. 2021 Jun 1;12(3):e00869-21. doi: 10.1128/mBio.00869-21 (PMC8262969; doi:10.1128/mBio.00869-21)
Supplement: TABLE S1 [file mbio.00869-21-st001.docx]

**Table S1** Number of OMVs/ml from various *Salmonella* deletion mutants

| Strains | **Number of OMV** $\boldsymbol{\times}$**10^11^ /ml*** | | | |
| --- | --- | --- | --- | --- |
|  | SL1344 *Salmonella* Typhimurium  (*fliC*::Cm^r^ *, fljB*::MudJ) | | | *Salmonella* Typhi |
| **Figure 1** | **A** | **B** | **C** | **D** |
| WT | 23 ± 0 | 20 ± 0.001 | 20 ± 0 | 0.11 ± 0.004 |
| Δ*pagC* | 3.2 ± 0.1 | - | 3.7 ± 0.15 | 0.07 ± 0.007 |
| Δ*pagC +* p*pagC* | - | - | 60.2 ± 0.42 | 0.09 ± 0.01 |
| Δ*phoP* | 3.8 ± 0.01 | - | - | - |
| Δ*pagP* | 5.3 ± 0.2 | - | - | - |
| Δ*pagN* | 15.3 ± 0.1 | - | - | - |
| Δ*pagL* | 22.5 ± 0.5 | - | - | - |
| Δ*pgtE* | 31.5 ± 0.3 | - | - | - |
| Δ*lpxO* | 23.4 ± 0.1 | - | - | - |
| Δ*pmrAB* | 16.5 ± 0.5 | - | - | - |
| PhoP^C^ | - | 29 ± 0.01 | - | - |
| PhoP^C^ Δ*pagC* | - | 1.1 ± 0.02 | - | - |
| Δ*phoP +* pRS1 | - | 1.1 ± 0.01 | - | - |
| Δ*phoP +* p*pagC* | - | 36 ± 0.04 | - | - |
| **Figure 2** | **A** | **B** | **C** |  |
| WT | 22 ± 0.02 | - | - |  |
| Δ*pagC* | 2.2 ± 0.01 | - | - |  |
| Δ*rck* | 21 ± 0.02 | - | - |  |
| Δ*ompX* | 23 ± 0.11 | - | - |  |
| Δ*pagC*Δ*rck*Δ*ompX*  + pRS1 | - | 4.7 ± 0.01 | - |  |
| Δ*pagC*Δ*rck*Δ*ompX*  + p*pagC* | - | 7.8 ± 0.01 | - |  |
| Δ*pagC*Δ*rck*Δ*ompX*  + p*rck* | - | 4.3 ± 0.04 | - |  |
| Δ*pagC*Δ*rck*Δ*ompX*  + p*ompX* | - | 4.7 ± 0.02 | - |  |
| Δ*pagC*Δ*rck*Δ*ompX*  + pMMB207 | - | - | 4 ± 0.01 |  |
| Δ*pagC*Δ*rck*Δ*ompX*  + p*ail* | - | - | 4.1 ± 0.05 |  |

* Normalized to 2 $\times$10^9^ cfu of bacteria /ml
