# Supplementary material for: Increased Production of Outer Membrane Vesicles by Salmonella Interferes with Complement-Mediated Innate Immune Attack
Source: mBio. 2021 Jun 1;12(3):e00869-21. doi: 10.1128/mBio.00869-21 (PMC8262969; doi:10.1128/mBio.00869-21)
Supplement: TABLE S3 [file mbio.00869-21-st003.docx]

**Table S3** List of bacterial strains, plasmids and primers.

| **Strains or Plasmids** | **Description** | **Antibiotics** | **Reference** |
| --- | --- | --- | --- |
| ***Salmonella* (or mutations in *Salmonella)*** | | | |
| SL1344 | *S. enterica* subp. *enterica* serovar Typhimurium (*S.* Typhimurium) | Str^r^ | (77) |
| LT2 *fliC* | *S.* Typhimurium *fliC*::Cm^r^ | Cm^r^ | Igor Brodsky |
| SL3201 | *S.* Typhimurium *fliC*::Tn10 *flj*B::MudJ | Tc^r^, Km^r^ | (78) |
| WT | SL1344 *fliC*::Cm^r^ *, fljB*::MudJ | Str^r^, Km^r^, Cm^r^ | This work |
| Δ*pagC* | *pagC* deletion in WT | Str^r^, Km^r^, Cm^r^ | This work |
| Δ*pagP* | *pagP* deletion in WT | Str^r^, Km^r^, Cm^r^ | This work |
| Δ*pagL* | *pagL* deletion in WT | Str^r^, Km^r^, Cm^r^ | This work |
| Δ*pagN* | *pagN* deletion in WT | Str^r^, Km^r^, Cm^r^ | (79) |
| Δ*phoP* | *phoP* deletion in WT | Str^r^, Km^r^, Cm^r^ | This work |
| Δ*rck* | *rck* deletion in WT | Str^r^, Km^r^, Cm^r^ | This work |
| Δ*ompX* | *ompX* deletion in WT | Str^r^, Km^r^, Cm^r^ | This work |
| Δ*pgtE* | *pgtE* deletion in WT | Str^r^, Km^r^, Cm^r^ | This work |
| Δ*lpxO* | *lpxO* deletion in WT | Str^r^, Km^r^, Cm^r^ | This work |
| Δ*pmrAB* | *pmrAB* deletion in WT | Str^r^, Km^r^, Cm^r^ | This work |
| Δ*pagC* Δ*rck* | *rck* deletion in Δ*pagC* | Str^r^, Km^r^, Cm^r^ | This work |
| Δ*pagC* Δ*ompX* | *pagC* deletion in Δ*ompX* | Str^r^, Km^r^, Cm^r^ | This work |
| Δ*pagC* Δ*rck* Δ*ompX* | *rck* deletion in Δ*pagC* Δ*ompX* | Str^r^, Km^r^, Cm^r^ | This work |
| Δ*pagC* Δ*rck* Δ*ompX* Δ*pgtE* | *pgtE* deletion in Δ*pagC* Δ*rck* Δ*ompX* | Str^r^, Km^r^, Cm^r^ | This work |
| ATCC 14028s *pho*-24 | *S.* Typhimurium *phoQ* constitutive mutant |  | (69) |
| PhoP^C^ | *phoQ_T48I_* (also designated *pho*-24) in WT | Str^r^, Km^r^, Cm^r^ | This work |
| PhoP^C^ Δ*pagC* | *pagC* deletion in PhoP^C^ | Str^r^, Km^r^, Cm^r^ | This work |
| *S.* Typhi | *S. enterica* subp. e*nterica* serovar Typhi 9,12,[Vi]:d:[Z66] |  | (79) |
| *S.* Typhi Δ*pagC* | *pagC* deletion in *S.* Typhi |  | This work |
| ***E. coli*** | | | |
| JKE201 | *E. coli* MG1655 RP4-2-Tc::[ΔMu1::Δaac(3)IV::lacI^q^-ΔaphA-Δnic35-ΔMu2::zeo] ΔdapA::(erm-pir) ΔrecA ΔmcrA Δ(mrr-hsdRMS-mcrBC)) |  | (67, 80) |
| **Plasmids** | | | |
| pOPC1-*pmrAB* | Allelic exchange plasmid to delete *pmrAB* | Km^r^ | (80, 81) |
| pMG81 | Expression plasmid with *tetR* and *tetA* promoter upstream of a cloned *envZ* gene, Ap^R^ | Amp^r^ | Mark Goulian |
| pRS1  (empty vector) | pMG81∆*envZ* | Amp^r^ | (79) |
| p*pagC* | SL1344 *pagC* in pRS1 | Amp^r^ | This work |
| p*rck* | SL1344 *rck* in pRS1 | Amp^r^ | This work |
| p*ompX* | SL1344 *ompX* in pRS1 | Amp^r^ | This work |
| pMMB207 | Expression plasmid with the tac promoter, Cm^R^ | Cm^r^ | ATCC^®^ 37809™, Addgene, (82) |
| p*ail* | *Yersinia pestis* *ail* in pMMB207 (pMMB207-ail) | Cm^r^ | (82) |
| **Primers** | **Sequence (5’ to 3’)** | | |
| *pagC*_Frag1.FOR | CCCAGTCTCGAGGTCGACGGTATCGATAAGCTTGATATCGggaattgtggtgttgattctattcttataatataacaagaaatg | | |
| *pagC*_Frag1.REV | gcttttcagaaacggtatcctgcataccccacggaaaaggc | | |
| *pagC*_Frag2.FOR | ccttttccgtggggtatgcaggataccgtttctgaaaagcataagct | | |
| *pagC*_Frag2.REV | CTGGAGCTCCACCGCGGTGGCGGCCGCTCTAGAACTAGTGgaaaaatctacaactcacaagtaataaaagacaataacatttttg | | |
| *pagP*_Frag1.FOR | CCCAGTCTCGAGGTCGACGGTATCGATAAGCTTGATATCGAAATGTACCGAATAAAGGATGGCGC | | |
| *pagP*_Frag1.REV | CCCCATCAAAACTGGAAACGCATCGCAACATACATACCG | | |
| *pagP*_Frag2.FOR | ACGGTATGTATGTTGCGATGCGTTTCCAGTTTTGATG | | |
| *pagP*_Frag2.REV | CTGGAGCTCCACCGCGGTGGCGGCCGCTCTAGAACTAGTGATTGGTCTGGATTGCAGAGAAGTGTAC | | |
| *pagL*_Frag1.FOR | cccagtctcgaggtcgacggtatcgataagcttgatatcgcacgtctgcgatatcctcggccc | | |
| *pagL*_Frag1.REV | cattgaaatggtggtggaagttgaataacaattagcgagttgctgg | | |
| *pagL*_Frag2.FOR | ccagcaactcgctaattgttattcaacttccaccaccatttcaatg | | |
| *pagL*_Frag2.REV | tggagctccaccgcggtggcggccgctctagaactagtgttattgttttcagcgcggaaatcgtcc | | |
| *phoP*_Frag1.FOR | CCCAGTCTCGAGGTCGACGGTATCGATAAGCTTGATATCGAGCTCGCGGACTTCCCGCGCCAGCG | | |
| *phoP*_Frag1.REV | actgttcttattgttaacacaataaatttgctcgccattttctgcc | | |
| *phoP*_Frag2.FOR | aaatggcgagcaaatttattgtgttaacaataagaacagtctagcgttga | | |
| *phoP*_Frag2.REV | CTGGAGCTCCACCGCGGTGGCGGCCGCTCTAGAACTAGTGGATCGATTTCGATCGCGATGTCTG | | |
| *lpxO*_Frag1.FOR | CCCAGTCTCGAGGTCGACGGTATCGATAAGCTTGATATCGatgcgcaggcagactttct | | |
| *lpxO*_Frag1.REV | CCCAGTCTCGAGGTCGACGGTATCGATAAGCTTGATATCGcagtctgtgaattacgccacgc | | |
| *lpxO*_Frag2.FOR | CTGGAGCTCCACCGCGGTGGCGGCCGCTCTAGAACTAGTGtaccgcgagaggagccg | | |
| *lpxO*_Frag2.REV | CTGGAGCTCCACCGCGGTGGCGGCCGCTCTAGAACTAGTGtgaaggcgaccataacatcgactg | | |
| *rck*_Frag1.FOR | CCCAGTCTCGAGGTCGACGGTATCGATAAGCTTGATATCGcggaagggaacgtgctgc | | |
| *rck*_Frag1.REV | cctgctctccgttatcagaatttcatgataaaactccctgaacacagttaagt | | |
| *rck*_Frag2.FOR | cagggagttttatcatgaaattctgataacggagagcaggaaagg | | |
| *rck*_Frag2.REV | CTGGAGCTCCACCGCGGTGGCGGCCGCTCTAGAACTAGTGatactttgtttcaggcatgtcttcagg | | |
| *ompX*_Frag1.FOR | CCCAGTCTCGAGGTCGACGGTATCGATAAGCTTGATATCGaacagcgcgaccgcc | | |
| *ompX*_Frag1.REV | tttcaccgacgtgattagaatttcataaccacctcaaatgtgcttcaag | | |
| *ompX*_Frag2.FOR | catttgaggtggttatgaaattctaatcacgtcggtgaaaatgaaaaatcc | | |
| *ompX*_Frag2.REV | CTGGAGCTCCACCGCGGTGGCGGCCGCTCTAGAACTAGTGaacgcgccgatgaagcgcatt | | |
| *pgtE*_Frag1.FOR | gtggtctagaagcgatactgagcatgttttttcatttctcttgtcctca | | |
| *pgtE*_Frag1.REV | CCCAGTCTCGAGGTCGACGGTATCGATAAGCTTGATATCGAATTCaatcgcctgccagtctctttc | | |
| *pgtE*_Frag2.FOR | CTGGAGCTCCACCGCGGTGGCGGCCGCTCTAGAACTAGTGGATCCggagatgtttgccaacgcga | | |
| *pgtE*_Frag2.REV | gagaaatgaaaaaacatgctcagtatcgcttctagaccacatcgg | | |
| PhoP^C^.FOR | CCCAGTCTCGAGGTCGACGGTATCGATAAGCTTGATATCGAAGAGCGGATCGATAAAGTTGCTAAAC | | |
| PhoP^C^.REV | CTGGAGCTCCACCGCGGTGGCGGCCGCTCTAGAACTAGTGGATCGATTTCGATCGCGATGTCTG | | |
| *pagC_Ty_*_Frag1.FOR | CCCAGTCTCGAGGTCGACGGTATCGATAAGCTTGATATCGCAAAATAGACTTTTTTATCGAGTGTTCAATATTTGCGT | | |
| *pagC_Ty_*_Frag1.REV | gcttttcagaaacggtatcctgcataccccacggaaaaggc | | |
| *pagC_Ty_*_Frag2.FOR | ccttttccgtggggtatgcaggataccgtttctgaaaagcataagct | | |
| *pagC_Ty_*_Frag2.REV | CTGGAGCTCCACCGCGGTGGCGGCCGCTCTAGAACTAGTGgaaaaatctacaactcacaagtaataaaagacaataacatttttg | | |
| p*pagC*.FOR | ACCACTCCCTATCAGTGATAGAGAAAAGTGCATGAAAAATATTATTTTATCCACTTTAGT | | |
| p*pagC*.REV | ATCCGCCAAAACAGCCATCAGAAACGGTATCCAACC | | |
| p*pagC_Ty_*.FOR | ACCACTCCCTATCAGTGATAGAGAAAAGTGCATGAAAAATATTATTTTATCCACTTTAGT | | |
| p*pagC_Ty_*.REV | TCCGCCAAAACAGCCATCAGAAACGGTATCCAATTCC | | |
| p*rck*.FOR | CCCCCCATGGTGATGAAAAAAATCGTTCTGTCC | | |
| p*rck*.REV | CCCCAAGCTTCAGAACCGGTAACCGACAC | | |
| p*ompX*.FOR | CCCCCCATGGTGATGAAAAAAATTGCATGTCTTTCAG | | |
| p*ompX*.REV | CCCCAAGCTTTAGAAGCGGTAACCTACGCC | | |
| qPCR_*pagC*.FOR | GAATCCGCTGGAGAATATCG | | |
| qPCR_*pagC*.REV | CAGAAACGGTATCCAACCC | | |
| qPCR_*rck*.FOR | TATGCCCAGAGCCGGATAGA | | |
| qPCR_*rck*.REV | CCAGTACGTCGCCTTCACAT | | |
| qPCR_*ompX*.FOR | TACGGTGTAGTCGGTGTG | | |
| qPCR_*ompX*.REV | TACGGCTCTGCTCGTAAG | | |
| qPCR_*rpoB*.FOR | ACGGTCGCGTATGTCCTATC | | |
| qPCR_*rpoB*.REV | GAGTTCGCCTGAGCGATAAC | | |
